# Biological Evaluation of Different Extracts of Aerial Parts of* Nepeta deflersiana* and Standardization of Active Extracts Using 8-Epi-7-Deoxyloganic Acid and Ursolic Acid by Validated HPTLC Method

**DOI:** 10.1155/2018/8790769

**Published:** 2018-09-13

**Authors:** Raha Orfali, Nasir Ali Siddiqui, Perwez Alam, Tawfeq Abdullah Alhowiriny, Areej Mohammad Al-Taweel, Sami Al-Yahya, Najwa Mohammed Majrashi, Rashad Mehmood, Shabana Iqrar Khan, Shagufta Perveen

**Affiliations:** ^1^Department of Pharmacognosy, College of Pharmacy, King Saud University, P.O. Box 2457, Riyadh 11451, Saudi Arabia; ^2^National Center for Biotechnology, Life Science and Environment Research Institute, King Abdulaziz City for Science and Technology (KACST), P.O. Box 6086, Riyadh 11461, Saudi Arabia; ^3^Department of Chemistry, University of Education, Vehari Campus, Vehari 61100, Pakistan; ^4^National Center for Natural Products Research, School of Pharmacy, University of Mississippi, 38677, USA

## Abstract

*Nepeta deflersiana *(Lamiaceae) is a well-known medicinal plant that grows in Saudi Arabia. This plant is used in Saudi and Yemeni folk medicine as an anti-inflammatory, carminative, and antirheumatic agent. In order to prove its use in folk medicine, four different extracts from the aerial parts of the plant: petroleum ether, chloroform, ethyl acetate, and* n*-butanol extracts were subjected to biological assays to screen PPAR*α* and PPAR_*ϒ*_ agnostic, antioxidant, anti-inflammatory, and cytotoxic activities. Ethyl acetate and* n*-butanol extracts of* N. deflersiana* NDEE and NDBE, respectively, showed a decrease in oxidative stress and inhibition of both NF-kB and iNOS activities with no cytotoxic effects on four human cancer cell lines. Both active extracts were standardized using two bioactive metabolites which were isolated from the aerial parts of the same plant [8-epi-7-deoxyloganic acid (compound** 1**) and Ursolic acid (compound** 2**)] by developing a validated HPTLC method. It was found to provide a sharp and compact band of compound** 1** at R_f_ = 0.07 and R_f_ = 0.57 for compound** 2**, using chloroform, methanol, and formic acid (8.9:0.8:0.3, v/v/v) as mobile phase at 550 nm. Compounds** 1** and** 2** were found in NDEE by 9.59 %, w/w, and 84.63 %, w/w, respectively, and by 11.97 %, w/w, and 21.26 %, w/w, respectively, in NDBE.

## 1. Introduction

**Figure 1:**
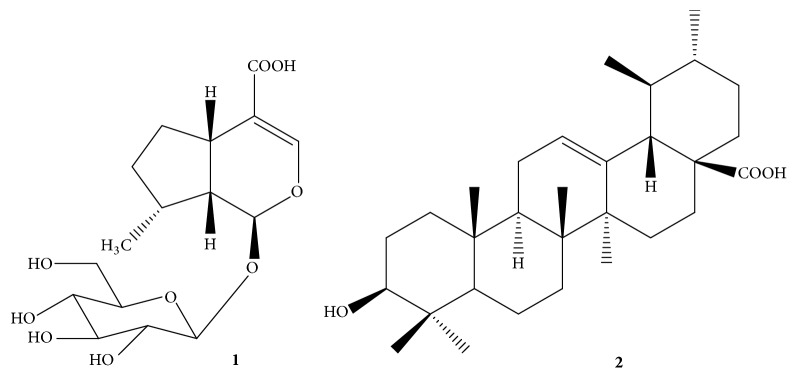
Chemical structure of biomarkers 8-epi-7-deoxyloganic acid** 1** and Ursolic acid** 2**.

*Nepeta* is one of the largest genera of the family Lamiaceae. It consists of 280 annual and perennial species [1] distributed widely in different regions including Asia, Africa, and southern parts of Europe [2]. Most of* Nepeta* species are used in folk medicine as an antiseptic for wounds, antispasmodic [3], antiasthmatic, diuretic [4], and carminative [5] as well as for the treatment of rheumatic disorders [6].* Nepeta deflersiana *Schweinf ex Hedge is the most intensively studied species in Saudi Arabia [7]. It was subjected to several biological investigations concerning its anticancer [8], antioxidant, anti-inflammatory, [9] antiviral, and antimicrobial activities [1] and cardioprotective properties [10]. In our previous phytochemical study on* N. deflersiana *growing in Saudi Arabia, we isolated several secondary metabolites from the ethanolic extract of aerial parts of the plant [11]. 8-Epi-7-deoxyloganic acid** 1** and Ursolic acid** 2** were the major constituents of this plant (Figure 1). Iridoid glycosides are reported to possess cardioprotective, hepatoprotective, immunomodulator, hypolipidemic, hypoglycemic, choleretic, and anti-inflammatory and antioxidant effects [12]. It was reported that the mechanism of anti-inflammatory effect of iridoid glycosides took place by inhibiting ROS production and/or exhibiting a radical scavenging effect during oxidative stress [13]. On the other hand, Ursolic acid is exhibiting a wide range of pharmaceutical properties as an anticancer agent as well as anti-inflammatory and antimicrobial properties; also it exhibits a protective effect on the liver, lungs, kidneys, and the brain [14]. A study showed the effects of Ursolic acid in improving the expression of PPAR*α* protein [15], and PPARs are ligand-stimulated transcription factors of the superfamily of a nuclear hormone receptor which consists of three subtypes: PPAR*α*, PPAR*γ*, and PPAR*β*/*δ*. This superfamily plays an important role in metabolic function and in energy homeostasis. PPAR-*α* decreases triglyceride levels when activated, whereas activation of PPAR-*γ* affects insulin sensitization and improves metabolism of glucose [16].

This study deals with a detailed biological analysis which includes the PPAR*α*, PPAR*γ* agonistic, antioxidant, anti-inflammatory, and cytotoxic effects of different extracts from the* N. deflersiana* leaves grown in Saudi Arabia. Those include petroleum ether, chloroform, ethyl acetate, and butanol extracts. Ethyl acetate and* n*-butanol extracts showed promising PPAR*α*, PPAR*γ* agonistic, antioxidant, and anti-inflammatory activities when compared to the other two extracts. Ethyl acetate and chloroform extracts showed moderate cytotoxic effect on noncancerous kidney cell lines (LLC-PK_1_).

Compounds** 1** and** 2** were chosen for standardization of their quantities in the two most active extracts, ethyl acetate NDEE and* n*-butanol extracts NDBE of the* N. deflersiana*, as mentioned above, for their potential anti-inflammatory and antioxidant activities.

Several chromatographic methods were reported for the quantification of iridoid glycosides and Ursolic acid, in different plant extracts. Those were by capillary high-performance liquid chromatography (capillary HPLC–ESI/MS) [17] and high-performance thin-layer chromatography (HPTLC), respectively [18], but a fully validated HPTLC method has not yet been reported for the quantification of 8-epi-7-deoxyloganic acid** 1** and Ursolic acid** 2** presented in* N. deflersiana*. Thus, the goal of the present study is to develop a validated HPTLC densitometric method for the comparative analysis of compounds** 1** and** 2** in the most active extracts: ethyl acetate NDEE and butanol NDBE extracts of* N. deflersiana* leaves grown in Saudi Arabia.

## 2. Experimental

### 2.1. Plant Material

The aerial parts of* N. deflersiana* (0.5 kg), with voucher specimen number (no. 15797), was collected from Abha in March 2014. The plant material was identified by Dr. Mohammad Atiqur Rahman, Professor of Taxonomy, College of Pharmacy, King Saud University, Saudi Arabia.

### 2.2. Extraction and Fractionation

The aerial parts of* N. deflersiana* (0.5 kg) were shade-dried, ground, and successively extracted at room temperature with EtOH: H_2_O (8:2, 3×3 Lit.). Four different fractions from the residue have been obtained: petroleum ether (5 g), chloroform (15 g), ethyl acetate (17 g), and* n*-butanol (13g). Isolation of compounds** 1** and** 2** has been described in our previous article [10].

The extract was evaporated to obtain the residue (100 g), which then was suspended in water and fractionated with solvents to obtain five fractions: petroleum ether (5g), chloroform (15 g), ethyl acetate (17 g),* n*-butanol (13 g), and the remaining water-soluble extract.

### 2.3. Biological Study

#### 2.3.1. PPAR*α* and PPAR_*ϒ*_ Activation

The activation of PPAR*α* and PPAR_*ϒ*_ was determined by a receptor gene assay in HepG2 cells transfected with pCMV-rPPAR_*ϒ*_ and pRREap2-tk-luc plasmids or pSG5-PPAR*α* and PPRE X3-tk-luc as reported before [19]. 96-well plates were seeded with transfected cells at a density of 5 X 10^4^ cells/well. The cells were exposed to different concentrations of test samples after 24 hours of incubation. Following that, luciferase activity was measured and the fold increase in luciferase activity in sample treated cells was calculated in comparison to vehicle treated cells. Rosiglitazone and ciprofibrate were used as drug controls in each assay.

#### 2.3.2. Inhibition of Cellular Oxidative Stress

The assay for cellular oxidative stress activity was carried out in HePG2 cells [20] as mentioned in our previous work [21]. In this assay the ABAP [2,2′-azobis (2-amidinopropane) dihydrochloride] is used to enhance intracellular generation of peroxyl radicals. HePG2 cells were seeded (60,000 cells/well) and incubated for 24 hours. The cells were washed with PBS cells treated with the test samples diluted in (25*µ*M DCFH-DA) for 1 hour. ABAP (600 *µ*M) was added to each well after removing the medium containing samples and the plate was immediately read on a Spectramax plate reader every 5 minutes for the duration of 1 hour (37°C, emission and excitation at 538 nm and 485nm, respectively). The positive control used in this test was quercetin. The antioxidant activity was calculated as below:

(1) %  decrease  in  oxidative  stress=100−AUC  sampleAUC  control  X  100.

#### 2.3.3. Inhibition of iNOS Activity

RAW264.7 mouse macrophage cell line was seeded in the wells at a density of 50,000 cells/well in 96-well plates and was left to grow for 24 hours for a confluency > 75%. Lipopolysaccharides (LPS,5 *µ*g/ml) were added after incubating with the test samples for 30 minutes and cells were incubated for 24 hours. Griess reagent was used to determine the nitric oxide (NO) level in the cell supernatant. The inhibition of NO production by the sample was detected in comparison to parthenolide control (Sigma-Aldrich, St Louis, MO, USA). Dose curves were used to calculate the IC_50_ value [19, 21].

#### 2.3.4. Inhibition of NF-KB Activity

96-well plates were used to seed human chondrosarcoma cells transfected with NF-KB luciferase plasmid at a density of 1.25 X 10^5^ cells per well. Cells were treated with various dilutions of test samples for 30 minutes after 24 hours' incubation. After that, the cells were induced with PMA (70 ng/ml) for 8 hours. Luciferase Assay Kit (Promegam Medison, WI, USA) was used for measuring luciferase activity. The decrease in luciferase expression indicates the inhibition of NF-KB activity. Parthenolide was used as positive control.

#### 2.3.5. Cytotoxicity

In this study the samples were tested against six different cell lines, four human cancer cell lines (SK-OV-3, SK-MEL, KB, and BT-549), and two noncancerous kidney cell lines (VERO and LLC-PK_1_) as reported earlier [22]. All cell lines were purchased from the American Type Culture Collection (ATCC, Rockville, MD). At a density of 25,000 cells/well cells were grown for 24 hours for confluency before adding the test samples and were then further incubated for 48 hours. The viability of cells was measured by Neutral Red assay according to Borenfreund et al. procedure [22]. Doxorubicin was used for positive control, whereas DMSO was used as negative control.

### 2.4. Quantitive Analysis

#### 2.4.1. Apparatus and Reagents

Analytical grade reagents and solvents (methanol, chloroform, and formic acid) were purchased from WINLAB and BDH (UK). The chromatograms were performed on a 10×10cm glass-precoated silica gel and the F_254_ HPTLC plates were procured from Merck (Darmstadt, Germany). The CAMAG Automatic TLC Sampler-4 (CAMAG, Switzerland) was used to apply standard and extract samples to HPTLC plates band. The ADC2 (automatic development chamber) (CAMAG, Muttenz, Switzerland) was used to develop the TLC plates and was documented by CAMAG TLC Reprostar 3. In order to scan the plates, CATS 4 (CAMAG) was used.

#### 2.4.2. Preparation of Standard Stock Solution

Standard stock solution of the iridoid glycoside 8-epi-7-deoxyloganic acid and Ursolic acid (1 mg/mL each) was made by dissolving 1 mg of standard in 1 mL methanol. Again, 1 mL of the stock solution was taken and 9mL methanol was added to it to make the final concentration of standard 100ng/*µ*L. For calibration, 1-10*µ*L of final standard solution was applied to normal phase plate to furnish the linearity range of 100-1000ng band^−1^.

#### 2.4.3. TLC Instrumentation and Chromatographic Conditions

The quantitative analysis of compounds** 1** and** 2** in* N. deflersiana* ethyl acetate extract (NDEE) and* N. deflersiana n*-butanol extract (NDBE) were carried out on 10 × 10 cm precoated Si gel 60 F_254_ glass plates. A microliter syringe was fitted with the Automatic TLC Sampler 4 (ATS4) (CAMAG) to apply different concentrations of compounds** 1** and** 2** along with different samples and standards on the TLC plates in 160nl/s application rate. Automatic developing chamber 2 (ADC2) was used to develop the plate in linear ascending mode with the following previously saturated mobile phase (For 20 min at 22°C) chloroform, methanol, and formic acid (8.9:0.8:0.3, v/v/v). The developed plates were dried and scanned at 515 nm wavelength by using the CAMAG TLC scanner IV with speed 20mm/s, noticing that the slit dimensions were 4.00 × 0.45 mm.

#### 2.4.4. Preparation of Calibration Graphs

Calibration graphs for standards iridoid glycoside 8-epi-7-deoxyloganic acid and Ursolic acid were prepared by spotting the standard with six different volumes in order to get different amounts of both isolates per spot. The height and the area were important parameters that have been taken into consideration versus the amount per spot.

#### 2.4.5. Method Development

Chromatograms were developed for compounds** 1** and** 2** by selecting the appropriate mobile phase after trying different combinations of solvents. The following mobile phase chloroform, methanol, and formic acid (8.9:0.8:0.3, v/v/v) have been selected according to the best resolution. This mobile phase has been subjected for the separation of both ethyl acetate and butanol extract. The mobile phase was saturated for 20 mins at 22°C at 515 nm wave length in absorbance mode by using the deuterium lamp.

#### 2.4.6. Method Validation

The proposed method was validated according to ICH guidelines for linearity range determination, precision, recovery as accuracy, limit of quantification (LOQ), limit of detection (LOD), and robustness [23].

#### 2.4.7. Linearity Range

A series of spots of several volumes (1 *µ*l-10 *µ*l) was applied in order to reach 100-1000 ng quantity of both compounds per band. The linearity range was statistically calculated using least square linear regression analysis from the graph which was plotted between the concentration and peak area.

#### 2.4.8. Precision

Replicate analysis (n=6) at three different concentration levels 200, 400, and 600ng/spot of compounds** 1** and** 2** was important to evaluate the precision (inter and intraday) of the proposed method. In order to detect the interday precision, the intraday assay should be repeated on three different days.

#### 2.4.9. Accuracy

Standard addition method was used to determine accuracy. The reanalyzed samples of** 1** and** 2** (200ng/spot) were spiked with the extra 0, 50, 100, and 150% of iridoid glycoside and Ursolic acid and the solutions were reanalyzed in six replicates (n=6) by the proposed method. The relative standard deviation (% RSD) percent recovery was then calculated.

#### 2.4.10. Robustness

Robustness was studied in triplicate at 300ng band^−1^ by making minor changes to mobile phase volumes, composition, and duration of saturation. The results were studied in terms of SD and % RSD of peak areas. Mobile phases prepared from chloroform: methanol: formic acid (8.9:0.8:0.3, v/v/v; 9:0.7:0.3 v/v/v; 8.8:0.9:0.3, v/v/v) were used for chromatography. The volume and duration of saturation of the mobile phase were investigated: 20 ± 2mL (18, 20, and 22 mL) and 20 ± 10min (10, 20 and 30 min), respectively. Before chromatography, the plates were activated at 110°C for 30 minutes.

#### 2.4.11. LOD and LOQ

The LOD is the lowest amount of an analyte that may be differentiated from the assay background at a distinct level of confidence and the LOQ is the minimum amount that can be quantified at a distinct level of precision or accuracy.

#### 2.4.12. Assay of Iridoid Glycoside and Ursolic Acid

Standard iridoid glycoside 8-epi-7-deoxyloganic acid and Ursolic acid along with test samples were spotted on HPTLC plates. The percentage of both compounds present in test samples NDEE and NDBE were determined by measuring the areas for the standard and test samples. Thus, the percentages of** 1** and** 2** were calculated for both samples of* N. deflersiana* species.

## 3. Results

### 3.1. Isolation of Compounds

**Figure 2:**
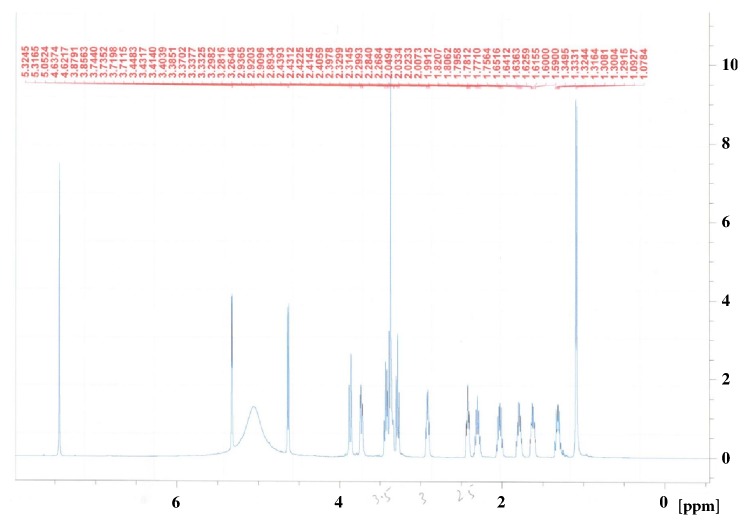
^1^H-NMR spectrum of** 1** (CD_3_OD, 500 MHz).

**Figure 3:**
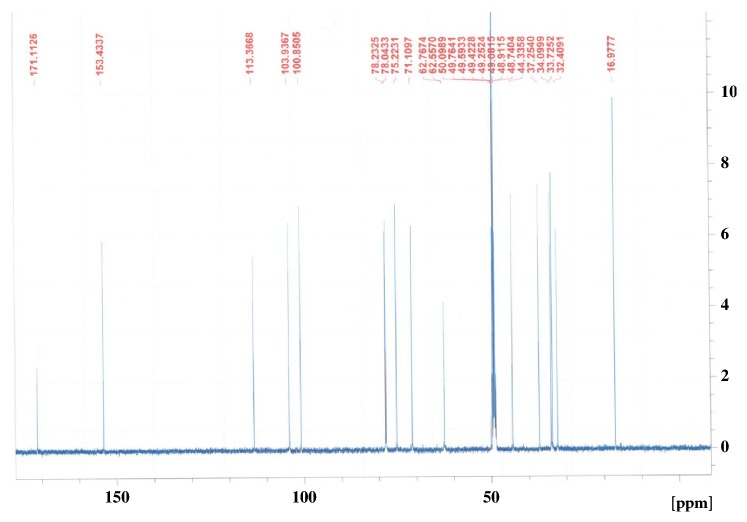
^13^C-NMR spectrum of** 1** (CD_3_OD, 125 MHz).

**Figure 4:**
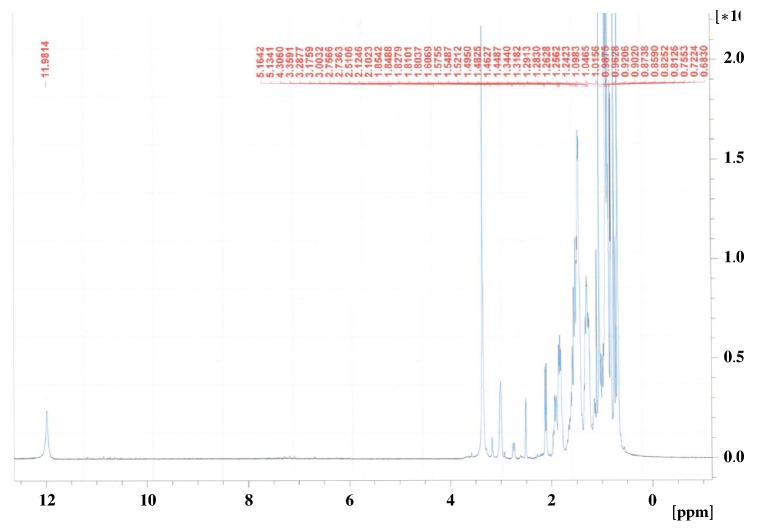
^1^H-NMR spectrum of** 2** (DMSO-*d*_6_, 700 MHz).

**Figure 5:**
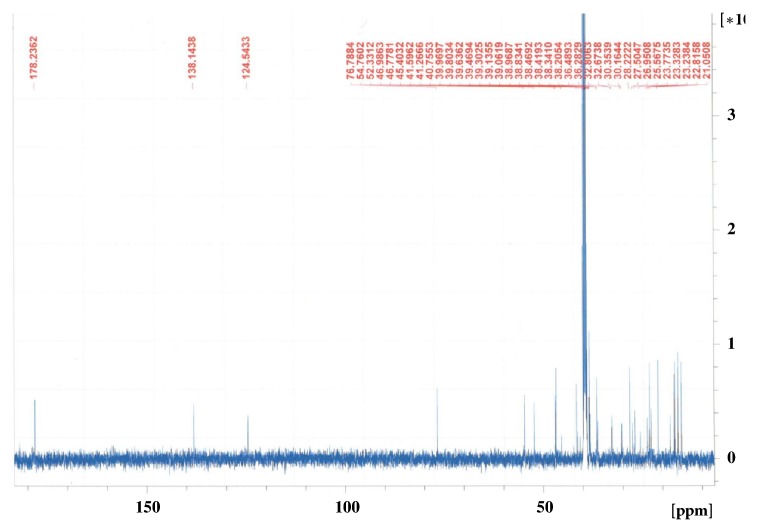
^13^C-NMR spectrum of** 2** (DMSO-*d*_6_, 175 MHz).

The ethyl acetate and* n*-butanol fraction of ethanol extract of the aerial parts of* N. deflersiana *were subjected to a series of column chromatographic separations to obtain compounds** 1** and** 2** (new in genus* Nepeta*). The structures of these compounds were established by MS and NMR spectroscopy (Figures 2–5) [11].

### 3.2. PPAR*α* and PPAR_*ϒ*_ Agonistic Activity

Four extracts of* N. deflersiana* were tested for their PPAR*α* and PPAR_*ϒ*_ agonistic activity and their results were represented in Table 1. Fold induction in the activity of PPAR was investigated in response to the extracts in comparison with untreated controls. A fold induction of 1.5 means a 50% increase in PPAR activation. Both extracts showed dual activation effects with promising activity. NDBE revealed better PPAR*α* and PPAR_*ϒ*_ specific agonistic activity than NDEE when compared to Rosiglitazone and Ciprofibrate 10 *µ*M, respectively. Petroleum ether and chloroform extracts did not show any PPAR agonistic activity.

### 3.3. Inhibition of Cellular Oxidative Stress, iNOS, and NF-kB Activities

Petroleum ether, chloroform, ethyl acetate, and butanol extracts of* N. deflersiana *aerial parts have been tested for their antioxidant and anti-inflammatory effects. Both extracts NDEE and NDBE showed promising cellular oxidative stress inhibitory activity and inhibition of NF-kB and iNOS activities (Table 2). NDEE showed higher decrease in cellular oxidative stress (55% decrease at 500 *µ*g/ml) than NDBE (52% decrease at 500 *µ*g/ml). The anti-inflammatory properties for both extracts listed in Table 2 were explained in terms of the inhibition of NF-kB and iNOS activities. NDEE was more active than NDBE and it inhibited the NF-kB activity with IC_50_ values of 17 *µ*g/ml while the inhibition by NDBE was at 39 *µ*g/ml. Interestingly, NDBE showed iNOS inhibition with IC_50_ 18 *µ*g/ml, more so than the NDEE, which showed inhibition with IC_50_ 29 *µ*g/ml. The petroleum ether and chloroform extracts did not show any anti-inflammatory activity.

### 3.4. Cytotoxic Activity

All four extracts were not cytotoxic against any of the four tested human cancer cell lines (SK-OV-3, SK-MEL, KB, and BT-549). In contrast, there is moderate cytotoxic action against one of the two tested noncancerous kidney cell lines (LLC-PK_1_) with IC_50_ of 16 and 55 *µ*g/ml for ethyl acetate and chloroform extracts, respectively (Table 3).

### 3.5. HPTLC Method Development and Validation

**Figure 6:**
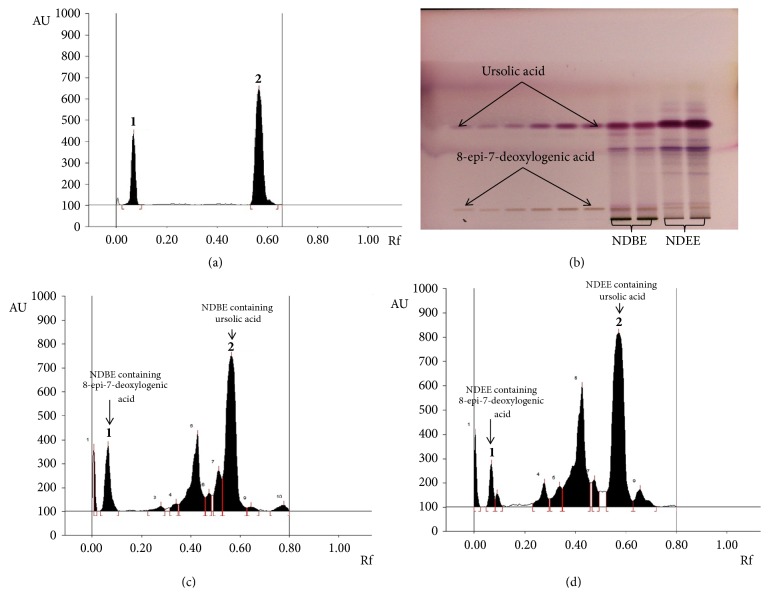
Quantification of 8-epi-7-deoxyloganic acid** 1** and Ursolic acid** 2 **in different fractions of* N. deflersiana* by HPTLC using chloroform, methanol, and formic acid (8.9:0.8:0.3, v/v/v) as mobile phase. (a) Chromatogram of standard** 1** (R_f_ = 0.07; 800 ng/spot) and** 2** (R_f_ = 0.57; 800 ng/spot) at *λ*max= 515 nm. (b) Pictogram of* p*-anisaldehyde derivatized TLC plate at day light. (c)* N. deflersiana n*-butanol extract [NDBE (**1**, spot 2, R_f_ = 0.07;** 2**, spot 8, R_f_ = 0.57)]; (b)* N. deflersiana* ethyl acetate extract [NDEE (**1**, spot 2, R_f_ = 0.07;** 2**, spot 8, R_f_ = 0.57)].

To obtain high resolution and reproducible peaks, mobile phases were selected by analyzing different compositions of a mixture of solvents. The desired profile was achieved in chloroform, methanol, and formic acid (8.9:0.8:0.3, v/v/v) at 550 nm and was found to be selective for development of** 1** and** 2** in NDEE and NDBE. The developed HPTLC method gave an intense, symmetrical, and compact peak of** 1 **and** 2**, respectively, R_f_= 0.07 and R_f_ = 0.57 (Figure 6).

Compounds** 1** and** 2** were validated by the correlation coefficient (r^2^) and linear regression equations were observed as Y= 4.128X+ 806.99 and 11.91X + 237.26, respectively. In the linearity range 100-1000 ng/spot for both compounds, a good linearity response for the developed method was revealed (Table 4). The mean values with ±SD of the slope were 4.128 ± 0.013 for compound** 1** and 11.91 ± 0.045 for compound** 2**, and intercept values were 806.99 ± 38.77 and 237.26 ± 20.58 for compounds** 1** and** 2**, respectively. The intraday and interday precision and accuracy for the assay of compounds** 1** and** 2** at three quality-control (QC) levels (200, 400, and 600 ng band^−1^) have been observed as well (Table 5). Intraday and interday precisions (n=6) for compound** 1** were found to be 1.24-1.45% and 1.18-1.45%, respectively, and 1.10-1.19% and 1.00-1.17% for compound** 2**, respectively, which demonstrated the good precision of the proposed method. Standard Deviation (SD) and relative standard deviation percent (% RSD) were also calculated at 300 ng band−1 for both compounds (Table 6). The samples fortified at four quality-control levels of both compounds resulted in good recoveries, by a percent range between 98.60 and 99.48% for compound** 1** and between 98.16 and 99.06% for compound** 2** (Table 5).

The limit of detection (LOD) and limit of quantification (LQD) for compound 1 were found to be 10.51 and 31.85 ng band^−1^, respectively, and 12.49 and 37.86 band^−1^ for compound 2, respectively (Table 4). This indicated that the proposed method exhibited a good sensitivity for the quantification of the above-mentioned compound.

Introducing minor deliberate changes in the duration of saturation, volume, and mobile phase composition used in the saturation of** 1** and** 2**, the low value of SD and % RSD indicates that the method was robust (Table 7).

### 3.6. Estimation of Compounds** 1** and** 2** in NDEE and NDBE

The utility of the validated HPTLC method was employed for the quantification of compounds** 1** and** 2** in NDEE and NDBE. These bioactive markers were found to exist in both extracts (Figure 2, C&D). NDEE was found to contain 9.59 *µ*g/mg (w/w) and 84.63 *µ*g/mg (w/w) of compounds** 1** and** 2**, respectively. On the other hand, NDBE was found to contain 11.97 *µ*g/mg (w/w) and 21.26 *µ*g/mg (w/w) of compounds** 1** and** 2**, respectively.

## 4. Discussion

The four extracted fractions from the aerial parts of the Saudi* N. deflersiana*; petroleum ether, ethyl acetate, chloroform, and n-butanol were subjected for different biological evaluations including antioxidant, anti-inflammatory, and PPAR*α*, PPAR*γ* agonistic activities.

This study indicated that NDEE fraction revealed promising PPAR*α* and PPAR_*ϒ*_ specific agonistic activity and that NDEE possessed a moderate effect when compared to Rosiglitazone and Ciprofibrate 10 *µ*M, respectively. The petroleum ether and chloroform extracts did not show any PPAR agonistic activity. These results are in agreement with the literature of the effect of Ursolic acid in improving the expression of PPAR_*α*_ activity [15] and our findings in which the ethyl acetate extract is rich in the above-mentioned compound. According to our knowledge, this could be considered as the first report on the PPAR agonistic activity of* N. deflersiana* extracts.

The NDEE showed antioxidant potential which is due to a higher decrease in cellular oxidative stress (55% decrease at 500 *μ*g/ml). The NDBE as well possesses potential antioxidant activity, but in intensity less than the ethyl acetate extract. It decreases the cellular oxidative stress by 52% decrease at 500 *μ*g/ml.

The data shown in Table 2 indicate that the anti-inflammatory properties of NDEE and NDBE could be described in terms of the inhibition of NF-kB's transcriptional activity and the inhibition of iNOS. However, the petroleum ether and chloroform extracts of the Saudi* N. deflersiana* species did not show anti-inflammatory activity, probably indicating the antagonistic action of various constituents present in them.

Regarding cytotoxic activity, only ethyl acetate and chloroform extracts showed moderate cytotoxic activity against tested (LLC-PK_1_) kidney cell lines with IC_50_ 16 and 55 *µ*g/ml, for ethyl acetate and chloroform extracts, respectively (Table 3). This is in agreement with the literature, which reported weak to moderate cytotoxic activity of* N. deflersiana* methanol extract [9].

Great research efforts have been recently undertaken to quantify pentacyclic triterpenoids and iridoid glycosides generally in different plant species [24, 25]. However, there is no full report on a validated HPTLC method for the quantification of 8-epideoxyloganic acid** 1** and Ursolic acid** 2** represented in Saudi* N. deflersiana*.

Ursolic acid is the major component of many traditional medicine herbs and has been reported to possess a wide range of biological functions, such as antioxidant, anticancer, and anti-inflammatory properties. Analysis of various literatures indicates that several effects of Ursolic acid are due to the inhibition of NF-*κ*B activity [14]. The iridoid glycoside 8-epi-7-deoxyloganic acid exhibits anti-inflammatory activity through inhibiting the production of ROS and/or displaying a radical scavenging effect during oxidative stress [13]. In our previous phytochemical study on* N. deflersiana* growing in Saudi Arabia, we isolated compounds** 1** and** 2** from several fractions. Compound** 1** existed in ethyl acetate,* n*-butanol, chloroform, and petroleum ether soluble fractions in good amounts, while compound** 2** existed only in ethyl acetate and* n*-butanol fractions [11]. Based on the biological importance of 8-epideoxyloganic acid** 1** and Ursolic acid** 2**, the present investigation aims at exploring the quantitative estimation of compounds** 1** and** 2** in the most biologically active* N. deflersiana *fractions.

In order to give better, sharp, and well defined peak resolution for both compounds in NDEE and NDBE, a good mobile phase was selected. The results of validated parameters such as correlation coefficient (r^2^), linear regression equations, intra- and interday precisions, standard deviation (SD), limit of detection (LOD), and limit of quantification (LQD) gave good indication of the proposed method. Eventually, this data proved the previously mentioned HPTLC quantitative study, which represents the good quantity of both compound** 1 **[9.59 *µ*g/mg (w/w)] and compound** 2 **[84.63 *µ*g/mg (w/w)] in NDEE and 11.97 *µ*g/mg (w/w) of compound 1 and 21.26 *µ*g/mg (w/w) of compound** 2** in NDBE fractions. Those could be responsible for anti-inflammatory, antioxidant, and PPAR*α*, PPAR*γ* agonistic activities, which is in agreement with the previously reported literatures about the wide range of pharmacological effects of both compounds [10, 11].

## 5. Conclusion

This work represents the first detailed antioxidative, anti-inflammatory, and PPAR*α*, PPAR*γ* agonistic investigation of four different extracts of* N. deflersiana* growing in the Kingdom of Saudi Arabia. The results revealed the promising antioxidant and anti-inflammatory activities of ethyl acetate and* n*-butanol extracts. 8-Epi-7-deoxyloganic acid** 1** and Ursolic acid** 2** were previously reported to have potential biological activities; therefore, both active extracts NDEE and NDBE were standardized using these compounds by developing a HPTLC method which is precise. A specific technique can be used for the exploration of both constituents in antioxidant and anti-inflammatory related genera of the plant kingdom. Ultimately, the overall biological investigation in this study justifies the use of* Nepeta* as an antidiabetic, antioxidant, and anti-inflammatory drug in folk medicines.

## Figures and Tables

**Table 1:** PPAR agonistic activity of *N. deflersiana* extracts.

Sample name	Fold induction					
	PPAR alpha			PPAR gamma		
	50 *µ*g/mL	25 *µ*g/mL	12.5 *µ*g/mL	50 *µ*g/mL	25 *µ*g/mL	12.5 *µ*g/mL
Pet. ether extract	NA	NA	NA	NA	NA	NA
CHCl_3_ extract	NA	NA	NA	NA	NA	NA
EtOAc extract	1.48	1.06	0.87	1.6	1.2	1.0
*n*-BuOH extract	1.86	2.03	1.74	1.9	1.7	1.2
Ciprofibrate 10 *µ*M	1.9	-	-	2.1	-	-
Rosiglitazone 10 *µ*M	2.2	-	-	2.6	-	-

NA= not active.

**Table 2:** Anti-inflammatory activity of *N. deflersiana* extracts.

^a^Sample Name	% decrease in oxidative stress	Inhibition of NF-kB activity IC_50_ in *µ*g/ml	Inhibition of iNOS activity IC_50_ in *µ*g/ml
Pet. ether extract	NA	NA	NA
CHCl_3_ extract	NA	NA	NA
EtOAc extract	55	17	29
*n*-BuOH extract	52	39	18
Parthenolide^b^	-	0.7	0.2
Quercetin^b^	74	-	-

^a^At 1000 *µ*g/ml.

^b^Positive control, NA: no activity up to 100 *μ*g/mL (NF-kB, iNOS) and 500 *μ*g/mL (oxidative stress).

**Table 3:** Cytotoxicity of *N. deflersiana* extracts.

Sample Name	Inhibition of cancer cells activity IC_50_ in *µ*g/ml				Inhibition of Non-cancer cells activity IC_50_ in *µ*g/ml	
	**SK-MEL**	**KB**	**BT-549**	**SK-OV-3**	**VERO**	**LLC-PK1**
Pet. ether extract	NA	NA	NA	NA	NA	NA
CHCl_3_ extract	NA	NA	NA	NA	NA	55
EtOAc extract	NA	NA	NA	NA	NA	16
*n*-BuOH extract	NA	NA	NA	NA	NA	NA
Doxorubicin	1.23	1.85	1.93	0.83	>5	0.85

NA= no cytotoxic activity up to 100 *µ*g/ml.

**Table 4:** R_f_, linear regression data for the calibration curve of 8-epi-7-deoxyloganic acid and Ursolic acid (n=6).

**Parameters**	8-epi-7-deoxyloganic acid **1**	Ursolic acid **2**
Linearity range (ng/spot)	100-1000	100-1000
Regression equation	Y= 4.128X+ 806.99	Y= 11.91X + 237.26
Correlation *(r*^*2*^) coefficient	0.9971	0.9955
Slope ± SD	4.128 ± 0.013	11.91 ± 0.045
Intercept ± SD	806.99 ± 38.77	237.26 ± 20.58
Standard error of slope	0.005	0.018
Standard error of intercept	15.82	8.40
R_f_	0.07 ± 0.0001	0.57 ± 0.04
LOD	10.51	12.49
LOQ	31.85	37.86

**Table 5:** Precision of the proposed HPTLC method (n=6).

Conc. of standard added (ng/spot)	8-Epi-7-deoxyloganic acid				Ursolic acid			
	Intraday Precision		Interday Precision		Intraday Precision		Interday Precision	
	Average Conc. found ± SD	%RSD	Average Conc. found ± SD	%RSD	Average Conc. found ± SD	%RSD	Average Conc. found ± SD	%RSD
200	198.18±2.47	1.24	193.33±2.29	1.18	198.84±2.19	1.10	197.16±2.09	1.06
400	396.67±5.19	1.30	391.82±5.01	1.27	396.91±4.61	1.16	395.23±4.49	1.13
600	595.55±8.69	1.45	590.70±8.33	1.41	597.76±7.17	1.19	595.24±7.01	1.17

**Table 6:** Recovery as accuracy studies of the proposed HPTLC Method (n=6).

(%) of 1 and 2 added to analyte	Theoretical conc. of 1 and 2 (*µ*g/mL)	Concentration found (*µ*g/mL) ± SD		%RSD		% Recovery	
		**1**	**2**	**1**	**2**	**1**	**2**
0	200	198.88 ± 2.35	196.32 ± 2.49	1.181	1.26	99.44	98.16
50	300	296.30 ± 3.77	295.22 ± 3.81	1.27	1.29	98.76	98.40
100	400	394.43 ± 5.61	396.24 ± 5.37	1.42	1.35	98.60	99.06
150	500	497.41 ± 7.39	494.06 ± 7.33	1.48	1.48	99.48	98.81

**Table 7:** Robustness of the proposed HPTLC Method (n=6).

Optimization condition	**1 **(300 ng/band)		**2 **(300 ng/band)	
	SD	%RSD	SD	%RSD
**Mobile phase composition;** **(chloroform: methanol: formic acid)**				
(8.9:0.8:0.3)	3.49	1.17	4.19	1.71
(9:0.7:0.3)	3.51	1.18	4.17	1.70
(8.8:0.9:0.3)	3.46	1.16	4.21	1.72
**Mobile phase volume** **(for saturation)**				
(18 mL)	3.55	1.19	4.23	1.72
(20 mL)	3.51	1.18	4.20	1.71
(22 mL)	3.49	1.17	4.25	1.73
**Duration of saturation**				
(10 min)	3.54	1.18	4.07	1.66
(20 min)	3.59	1.20	4.03	1.64
(30 min)	3.51	1.18	4.09	1.67

## Data Availability

The data used to support the findings of this study was included within the article and prior studies were cited at relevant places within the text as references [11].
